# Epidemiology of plasmid-mediated quinolone resistance in *salmonella enterica *serovar typhimurium isolates from food-producing animals in Japan

**DOI:** 10.1186/1757-4749-2-17

**Published:** 2010-12-07

**Authors:** Tetsuo Asai, Chizuru Sato, Kaori Masani, Masaru Usui, Manao Ozawa, Tomoe Ogino, Hiroshi Aoki, Takuo Sawada, Hidemasa Izumiya, Haruo Watanabe

**Affiliations:** 1 National Veterinary Assay Laboratory, Ministry of Agriculture, Forestry and Fisheries, 1-15-1 Tokura, Kokubunji, Tokyo 185-8511, Japan; 2 Nippon Veterinary and Life Science University, 1-7-1 Kyonancho, Musashino, Tokyo 180-8602, Japan; 3 National Institute of Infectious Diseases, 1-23-1 Toyama, Shinjuku-ku, Tokyo 162-8640, Japan

## Abstract

A total of 225 isolates of *Salmonella enterica *serovar Typhimurium from food-producing animals collected between 2003 and 2007 were examined for the prevalence of plasmid-mediated quinolone resistance (PMQR) determinants, namely *qnrA, qnrB, qnrC, qnrD, qnrS, qepA *and *aac(6')Ib-cr*, in Japan. Two isolates (0.8%) of *S*. Typhimurium DT104 from different dairy cows on a single farm in 2006 and 2007 were found to have *qnrS1 *on a plasmid of approximately 9.6-kbp. None of the *S*. Typhimurium isolates had *qnrA, qnrB, qnrC, qnrD*, *qepA *and *acc(6')-Ib-cr*. Currently in Japan, the prevalence of the PMQR genes among *S*. Typhimurium isolates from food animals may remain low or restricted. The PFGE profile of two *S*. Typhimurium DT104 isolates without *qnrS1 *on the farm in 2005 had an identical PFGE profile to those of two *S*. Typhimurium DT104 isolates with *qnrS1*. The PFGE analysis suggested that the already existing *S*. Typhimurium DT104 on the farm fortuitously acquired the *qnrS1 *plasmid.

## Findings

*Salmonella enterica *serovar Typhimurium is prevalent in many animal species [1-3] including food-producing animals that are considered to be reservoirs for human infection. *S*. Typhimurium was the top 5 serovar found most frequently in cases of *Salmonella *foodborne illness in Japan between 2006 and 2010 https://hasseidoko.mhlw.go.jp/Byogentai/Pdf/data48e.pdf. Multidrug-resistant *S*. Typhimurium definitive phage type 104 (DT104) causes human salmonellosis in Japan [3]. *S*. Typhimurium DT104 was first isolated in the late 1980 s, and has spread widely among food-producing animals across Japan [3-5]. Although a decreased proportion of DT104-related isolates among the animals was found between 2002 and 2005, multidrug-resistant *S*. Typhimurium remains prevalent among food-producing animals in Japan [6].

In Japan, fluoroquinolone drugs were approved in veterinary fields in 1991 and are commonly used for treatment of bacterial diseases such as enteritis and pneumonia in food-producing animals [7]. In 2001, fluoroquinolone resistance was found in *S*. Choleraesuis from pigs [8] and *S*. Typhimurium from cattle [9]. In addition, a fluoroquinolone-resistant *S*. Typhimurium was identified in bovine isolates in 2005 [6]. The mechanism of fluoroquinolone resistance in these isolates is the mutation of quinolone resistance-determining regions (QRDRs) in DNA gyrase and topoisomerase IV [8,9]. In 2006, *qnrS1 *was identified in two *S*. Typhimurium isolates (including one DT104 isolate) from dairy cows and beef cattle, and *S*. Thompson from poultry in Japan [10]. The report identified the potential risk of foodborne infections of *Salmonella *conferring the gene from food-producing animals to humans in Japan.

Quinolone resistance mechanisms mediated by plasmids are responsible for target protection such as the *qnr *genes, active efflux such as *qepA*, and enzymatic modifications such as *aac(6')Ib-cr *[11]. The plasmid-mediated quinolone resistance (PMQR) genes contribute to a reduction of quinolone susceptibility. In Japan, *qnrS *was first identified in human isolates of *Shigella flexneri *in 2003 [12]. *qepA*-harboring clinical isolates of *Escherichia coli *were found in 2002 in Japan [13]. *qnrB *in *Klebsiella oxytoca*, *Pseudomonas mirabilis*, and *P. fluorescens*, and *qnrS *in *E. coli *and *Enterobacter cloacae *were found in zoo animal isolates in 2006 [14]. In addition, the presences of *qnrS1 *and *qnrS2 *in *Salmonella *isolated from fecal samples of overseas travelers were reported in Japan [15]. These reports provided an infectious source of *Enterobacteriaceae *conferring plasmid-mediated quinolone resistance in Japan. We examined the prevalence of plasmid-mediated quinolone resistance in *S*. Typhimurium isolated from food-producing animals.

A total of 225 isolates of *S*. Typhimurium from food-producing animals collected between 2003 and 2007 were derived from 156 cattle, 62 pigs and 7 poultry: includes 42 isolates of DT104, 8 of DT104B, and 2 of U302 (Table 1). Bacteriophage typing was performed according to the methods of the Health Protection Agency, London, United Kingdom [16]. Of the isolates, 132 *S*. Typhimurium isolates collected between 2003 and 2005 [6] were subjected to detection of the PMQR genes. The remaining 93 isolates between 2006 and 2007 were investigated for the presence of the PMQR genes and antimicrobial susceptibility. The presence of *qnrA*, *qnrB *and *qnrS *genes was determined by PCR [17]. The *qnrC *and *qnrD *genes were detected using the primers as previously described [18,19], respectively. The *qepA *and *acc(6')-Ib-cr *genes were examined as previously described [20,21]. Nucleotide sequences of both strands were determined directly on PCR products. The DNA alignments and deduced amino acid sequences were examined using the BLAST program (National Center for Biotechnology Information, USA). Minimum inhibitory concentrations (MICs) of antimicrobial agents were determined using the agar dilution methods according to the Clinical and Laboratory Standards Institute (CLSI) guidelines [22]. The following 11 antimicrobials were tested: ampicillin (ABPC), cefazolin, colistin, chloramphenicol (CP), dihydrostreptomycin (DSM), gentamicin, kanamycin, oxytetracycline (OTC), nalidixic acid, enrofloxacin (ERFX), and trimethoprim. The MICs of each antimicrobial agent were interpreted using the recommendations of the CLSI [23]. The breakpoints not seen in the CLSI were defined in a previous study [1]. *Staphylococcus aureus *ATCC 29213, *Enterococcus faecalis *ATCC29212, *E. coli *ATCC 25922 and *P. aeruginosa *ATCC 27853 were used as quality control strains.

**Table 1:** Salmonella Typhimurium isolates used in this study

	Cattle				Pig			Poultry	
				
Isolation		Phagetype				Phagetype			Phagetype
							
year	Typhimurium	104	104B	U302	Typhimurium	104	104B	Typhimurium	104
2003	24	8	2	0	8	0	0	0	0
2004	25	3	0	2	8	1	0	0	0
2005	42	12	0	0	21	1	0	4	1
2006	23	4	0	0	11	2	0	2	1
2007	42	4	4	0	14	5	2	1	0

Total	156	31	6	2	62	9	2	7	2

Of 225 *S*. Typhimurium isolates, two isolates of DT104, 18-PLS-16 and 19-PLS-45, from different dairy cows on a single farm in 2006 and 2007 showed *qnrS *positive results. The sequencing of amplicons showed complete identity to *qnrS1 *previously identified on pAH0376 from a *S. flexneri *strain. None of the *S*. Typhimurium isolates had *qnrA, qnrB, qnrC, qnrD*, *qepA *and *acc(6')-Ib-cr*. The two isolates exhibited ERFX resistance (ERFX MIC, 2 mg/L) with resistances to ABPC, DSM, OTC and CP (Table 2).

**Table 2:** Susceptibility for several fluoroquinolones

Antimicrobials	18-PLS-16	19-PLS-45	17-PLS-75
Year isolated	2006	2007	2005
Sources	Cattle	Cattle	Cattle
qnr	qnrS1	qnrS1	-
mutation in gyrA	WT	WT	S83F&D87N
mutation in parC	WT	WT	S80R
phagetype	104	104	12

Naldixic acid	32	32	256
Oxolinic acid	4	4	>64
Flumequine	16	8	>64
Benofloxacin	4	4	16
Ciprofloxacin	1	1	8
Danofloxacin	2	2	16
Difloxacin	8	4	>32
Enrofloxacin	2	2	16
Levofloxacin	1	1	8
Norfloxacin	2	2	16

The QRDR of *gyrA*, *parC *and *parE *was examined in ERFX-resistant isolates by PCR amplification and sequencing using primers as described elsewhere [24]. In addition, susceptibility of ERFX-resistant isolates to fluoroquinolones was examined using the micro broth dilution methods according to CLSI guidelines [22]. For evaluation of active efflux of the ERFX-resistant bacteria, the MIC of ERFX was determined by the agar dilution method in the presence of carbonyl cyanide *m*-chlorophenylhydrazone (CCCP) (100 μM). They had no mutations in the QRDR of GyrA, ParC and ParE. The MIC of ERFX was not changed in the presence of CCCP (100 μM). The two isolates with *qnrS1 *exhibited almost the same MIC observed for each fluoroquinolone, which is relative low compared with the MIC for isolate (17-PLS-75) with mutations in the QRDR of GyrA and ParC.

**Figure 1:**
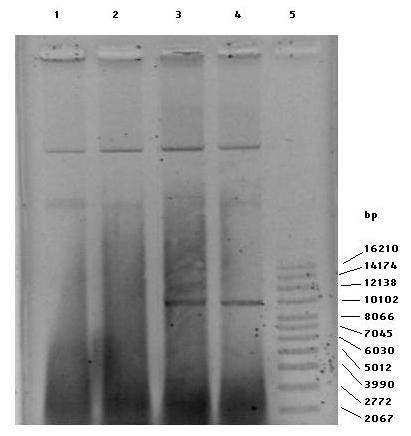
**Plasmid profile of *S.* Typhimurium isolates on a farm**. Lane 1: 17-PLS-27, Lane 2: 17-PLS-28, Lane 3: 18-PLS-16, Lane 4: 19-PLS-45, Lane 5: Super coiled DNA ladder.

Plasmid DNA was isolated from the *qnrS1*-positive isolates by the alkaline lysis method [25]. Extracted plasmids were transferred to Hybond-N+ membrane (Amersham Biosciences, Buckinghamshire, UK) using capillary blotting apparatus. The *qnrS1 *PCR product was labeled with DIG-11-dUTP by PCR using a DIG High Prime DNA Labeling Kit (Roche Diagnostics Ltd, East Sussex, UK). After hybridization with the *qnrS1 *probe, hybridized DNA was detected using a DIG Nucleic Acid Detection Kit (Roche Diagnostics Ltd). Using a plasmid profiling test, an approximately 93-kbp plasmid (virulence plasmid) was found in all four isolates, whereas there was also an approximately 9.6-kbp plasmid found in the *qnr*-conferring isolates. Hybridization tests revealed that *qnrS1 *was located on the 9.6-Kbp plasmid (Figure 1).

**Figure 2:**
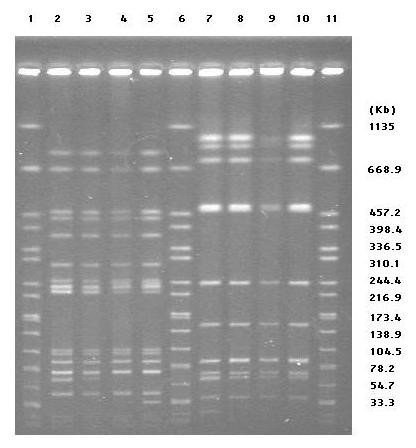
**PFGE profile of *S*. Typhimurium isolates on a farm**. Electrophoresis was performed using a CHEF-DR III System (Bio-Rad Laboratories, Hercules, CA, USA) with running conditions of 1 phase from 2.2 to 63.8 s at 180 V for 19 h. Lane1, 6, 11: *Salmonella* Braenderup H9812 digested with XbaI. Lane 2 and 7: 17-PLS-27, Lane 3 and 8: 17-PLS-28, Lane 4 and 9: 18-PLS-16, Lane 5 and 10: 19-PLS-45. Lane 2 to 5: XbaI digestion, Lane 7 to 10: BlnI digestion.

The appearance of *S*. Typhimurium DT104 conferring *qnrS1 *on the farm is caused either by the introduction of *S*. Typhimurium DT104 conferring *qnrS1 *or the transfer of the *qnrS1 *plasmid to *S*. Typhimurium DT104 already existing on the farm. According to the CDC PulseNet protocol [26], genetic relatedness of isolates were analyzed by PFGE with XbaI and BlnI restriction enzymes. The isolates tested included two *qnrS1*-negative isolates of *S*. Typhimurium DT104 isolated in 2005 on a farm in which *qnrS1*-conferring isolates were found. In the present study, it was difficult to precisely distinguish between the two *S*. Typhimurium DT104 isolates without *qnrS1 *and the two *S*. Typhimurium DT104 isolates with *qnrS1 *by PFGE analysis (Figure 2). Our previous study showed that there is a variation in the BlnI-digested PFGE profiles of *S*. Typhimurium DT104 isolated from food-producing animals in Japan [5]. These results suggested that the *S*. Typhimurium DT104 already present on the farm fortuitously acquired the *qnrS1 *plasmid. Previous studies showed that *qnrS1 *in Typhimurium isolated in the UK was present on plasmids of 10,066 bp, which were transferable by the conjugation test and carry an IncN replicon [27,28]. Further study need to clarify the source of plasmid bearing *qnrS1*.

This study demonstrated that the two isolates of *S*. Typhimurium collected from different cattle on a farm in 2006 and 2007 harbored *qnrS1 *on a 9.6-Kbp plasmid. At present in Japan, dissemination of *qnrS1 *among *S*. Typhimurium isolates from food animals may remain restricted. The spread of plasmids carrying *qnr *among *Salmonella *isolates of animal origin could have serious consequences for fluoroquinolone treatment of non-typhoid *Salmonella *infection in humans and animals. Previously, *qnrS1 *and *qnrS2 *were found in serovars Typhimurium, Corvallis, Montevideo, Agona, Braenderup and Alacua of *Salmonella *isolates from fecal samples of overseas travelers who had visited Thailand, Malaysia, Vietnam, Indonesia and Singapore, between 2001 and 2007 [15]. PMQR is identified in human isolates of *Enterobacteriaceae *but is likely to be rare in isolates from food-producing animals [29]. However, in China, plasmid-mediated quinolone resistance is frequently found in the isolates from food-producing animals [20]. Thus it would be difficult to prevent the invasion of resistance genes from foreign countries to Japan. The monitoring of fluoroquinolone use and quinolone resistance in bacteria of food-producing animal origin is essential to assess the level of risk of resistance in food-borne bacteria in the animals.

## Abbreviations

ABPC: ampicillin; CCCP: carbonyl cyanide *m*-chlorophenylhydrazone; CLSI: Clinical and Laboratory Standards Institute; CP: chloramphenicol; DSM: dihydrostreptomycin; DT104: definitive phage type 104; MICs: Minimum inhibitory concentrations OTC: oxytetracycline; PMQR: plasmid-mediated quinolone resistance; QRDRs: quinolone resistance-determining regions; ERFX: enrofloxacin.

## Competing interests

The authors declare that they have no competing interests.

## Authors' contributions

TA conceived the study, the study design, participated in the determination of quinolone resistance and determinants, interpreted the data and drafted the manuscript. CS carried out large parts of the experimental work. KM helped to carried out prevalence of resistance genes. MU helped to carried out prevalence of resistance genes. MO carried out the antimicrobial susceptibility testing. TO carried out the antimicrobial susceptibility testing. HA helped to carry out determination of quinolone resistance and draft the manuscript. TS helped to draft the manuscript. HI carried out phage typing and helped to draft the manuscript. WH helped to draft the manuscript. All authors read and approved the final manuscript.
